# Spatial variations and health risk assessment of heavy metal levels in groundwater of Qatar

**DOI:** 10.1038/s41598-024-64201-6

**Published:** 2024-07-10

**Authors:** Yehia Manawi, Mosab Subeh, Jaber Al-Marri, Huda Al-Sulaiti

**Affiliations:** Qatar Environment and Energy Research Institute, Hamad Bin Khalifa University, Qatar Foundation, P.O. Box 34110, Doha, Qatar

**Keywords:** Brackish water, Hazard index, Trace elements, Health effects, Environmental impact, Natural hazards

## Abstract

The present work’s objective is to give a comprehensive overview of the quality of groundwater in Qatar in terms of heavy metals content as well as investigating the cause and effect of the elevation in their levels above the WHO/US-EPA standards. The scope of the study included (1) physical and chemical analysis of 82 groundwater samples collected from various locations around Qatar, (2) development of ArcGIS maps depicting the variations in the levels, (3) assessment of the human health risks associated with the existing levels using three of the most used models which are: Hazard index (HI), Nemerow comprehensive pollution index (NCPI) and Incremental Lifetime Cancer Risk (ILCR). There is no extensive study ever reported to assess the health risks linked with the consumption of groundwater characterized with such heavy metals levels in Qatar. The chronic daily intake (CDI) of the investigated heavy metals (Ag, Mn, Cr, V, Mo and Sr) through ingestion and dermal pathways had a range of 1.4 × 10^–5^–6.7 × 10^–1^ mg/kg/day while the NCPI’s range was reported at 0–4.39. Moreover, the HI and ILCR were found to have a range of 0–3.2 and 5.6 × 10^–4^–5.5 × 10^–2^, respectively. The assessment of health risks, conducted in the present work, could be beneficial in building the baseline of heavy metals levels in groundwater in Qatar. This will also help in the determination of any future contamination of groundwater.

## Introduction

Qatar is a peninsula positioned in the Arabian Gulf bordering Saudi Arabia to the south. Despite the fact that Qatar is one of the richest nations in the world with a GDP of around 67,000 USD per capita, it is considered as one of the poorest nations in terms of renewable freshwater resources. The available renewable freshwater resources in Qatar were estimated at 33 m^3^ per year per capita whereas countries with available freshwater resources below 500 cubic meter are experiencing absolute water scarcity according to Falkenmark’s indicator of water stress^1,2^. The water scarcity in Qatar can be attributed to the low rain fall estimated at 78 mm per year and the over abstraction of groundwater^3^. The annual safe yield of groundwater in Qatar in 2021 was estimated at 54.2 million m^3^ by the planning and statistics authority (PSA) in Qatar, whereas the existing annual abstraction rates is 250 million cubic meter^4,5^. Moreover, the annual extraction rate of groundwater in Qatar in 2009 was reported by Alhaj^6^ to be around 444 million m^3^ or seven times the annual safe yield. The over pumping of groundwater in Qatar has not only reduced the quantity of groundwater but also their quality. The area underlain by groundwater with low TDS (< 3000 ppm) was reported by Schlumberger Water Services to drop significantly between 1972 and 2009^7^. Moreover, the urbanization and the growth in the industrial and economic sectors in Qatar have accelerated the groundwater contamination rates by not only natural but also man-made sources such as chemical and physical contamination sources. The chemical contamination of groundwater by oil was well investigated in literature^8–10^. For instance, the presence of polycyclic aromatic hydrocarbons (PAH) in Tigris River in Iraq was reported to arise from the attacks on oil refineries which elevated human health risks^9^. Moreover, the level of PAH in Euphrates River was reported to originate from the combustion of petroleum products which elevated not only the level of PAH but also their human health risks^10^. Furthermore, the evaluation of the level of PAH in Danube River (Hungary) was studied and found to be affected by wastewater and industrial processes taking place along the river. A PAH level of 365.8 ng/L was observed in the water samples collected from the river over the period of 12 months. These levels were reported to be higher than the allowable limit of 100 ng/l with a domination of pyrogenic sources^8^. Moreover, oil tank leakage was reported by US Environmental Protection Agency’s (USEPA) in 2011 to take place in 71% of all the buried oil tanks in USA that are older than 10 years^11,12^. The chemical contamination of groundwater does not include oil contamination merely but also heavy metals which may reach groundwater from industry in Qatar. Similarly, the overuse of pesticides in agriculture was reported to contaminate groundwater by various toxic and persistent compounds. Strontium is essential in growth and development of our bones and in the inhibition of osteoporosis^13^. The total daily intake of Sr as reported by the world health organization is 4 mg of which 0.7–2 mg comes from drinking water while 1.2–2.3 comes from food^14^. The elevation of Sr levels in human body has various adverse health effects on humans as Sr has the ability to mimic calcium within our bodies^15^. It was reported that elevated levels of Sr has the ability to impair the growth of bones in children; hence, children are more prone to Sr health effects than adults^16^. There is no limit or standard for Sr in drinking water reported by the World Health Organization (WHO)^17^. In 2012, the US EPA reported the recommended Sr level for lifetime health at 4 mg/L in their drinking water standards as well as health advisories report^18^. Two years later, the US EPA lowered the health reference level of Sr to 1.5 mg/L^19,20^.

Metal ions are extremely essential for living cells as they help in maintaining the lifespan of humans, animals and plants. However, metal ions can be toxic to humans and other living organisms. There are more than 50 elements which are known as heavy metals; 17 of these metals are very toxic and can be accessed by humans^21,22^. The level of toxicity of any metal is largely dependent on the type of metal itself as well as the biological role^21^. Such metals can be transferred to humans by the biomagnification process which tends to elevate the level of a particular substance in an organism to a higher level as we go up in the food chain. The most toxic heavy metals which are found in groundwater and can cause poisoning are: chromium, copper, cadmium lead, zinc and iron^21^. The exposure to lead from drinking water accounts for 20% of the average adult’s total exposure to lead. Exposure to lead from drinking water was reported to cause memory loss, lack of concentration, depression, kidney’s damage, reduced sperm count, spontaneous miscarriages as well as various nervous system damages. Arsenic on the other hand is also toxic to humans and was reported to increase the rate of cancer in kidney, liver, bladder and lung as well as significant damages to the nervous system.

Nemerow comprehensive pollution index (NCPI) is one of the most reported models in literature which are widely used to assess the quality of various environmental elements such as: groundwater^23^, surface water^24^, soil^25^, air^26^, etc. NCPI takes into consideration not only the effect of all of the investigated pollutants in the study but also emphasizes the pollutants that show elevated levels. The assessment of the pollution of groundwater with various pollutants such as trace metals using NCPI method was also reported in literature^23,27–30^. For instance, the investigation of the pollution of groundwater with heavy metals (Pb, Cr(VI), Se, Cd, As, Mn, Cu, and Zn) in the northwestern part of China was carried out by Mamat^29^. They found out that the NCPI of the studied samples had a range of 0.1–3.01 and a mean of 0.67. NCPI values greater than 3 was reported in literature to refer to environmental systems with high pollution content^31^. Likewise, Zhang^28^ assessed the pollution of groundwater with heavy metals (Ni, Cd, Cr, Cu and Zn) by analyzing 69 samples collected from farmlands in Huaibei area (China). The estimated NCPI ranged between 0.2 and 9.3 with an average of 1.9. The estimated NCPI of the analyzed groundwater was reported to show an extremely poor pollution level with Cr being the highest contributor to the health risks caused by heavy metals. In the present study, NCI will be used to evaluate the quality of groundwater in Qatar by investigating the pollution of groundwater with trace metals using a comprehensive pollution assessment method.

The conventional method used for the evaluation of the health effects of heavy metals involved comparing the existing levels with the reference levels; however, this method is not accurate as it does not take into consideration various factors such as: type of heavy metals present, exposure rates, body weight, etc. The carcinogenic human health risks can be evaluated by the determination of the US EPA’s Incremental Lifetime Cancer Risk (ILCR) which aims to correlate the effect of environment on the human health as well as quantify the degree of hazards present in in environment. This method takes into consideration all the potential pathways such as ingestion and dermal despite the fact that ingestion is the largest contributor to the human body. The investigation of the cancer risks associated with the exposure to heavy metals in groundwater used for drinking in Nigeria showed an ILCR as high as 48.5 in some urban areas^32^. The elevated levels of heavy metals in drinking water resources must attract the attention of Nigerian government to take actions in reducing their levels and consequently their health effects. Moreover, another study was conducted to assess the quality of groundwater in some parts of Tamil Nadu in India and also the carcinogenic health risks. It was reported that ILCR corresponding to the groundwater ranged between 0.013 and 0.052 which was significantly greater than the level recommended by US EPA of 1 1 × 10^–6^. Grmasha^9^ studied the level and health effect of polycyclic aromatic hydrocarbons (PAH) present in Tigris river as a result of the destruction of nearby oil refineries following the war in Iraq. The PAH level was observed to be as high as 3750 ng/L while the incremental lifetime cancer risk was high risk with reported adverse health effects such as cancer. Moreover, the assessment of the human health risks associated with presence of PAH in Euphrates River was reported to show an ILCR 10^–3^–10^–2^ which was observed to be up to 6 times the levels mentioned in literature^10^. Such levels and their associated risks were reported by the authors to call the environmental authorities in Western Asia for urgent attention^10^. Furthermore, the elevated PAH in Danube River which was observed to be 265% higher than the allowable limit of 100 ng/l, resulted in an ILCR above 10^–4^ for children and adults^8^.

The investigation of the level of chemical parameters in the groundwater is critical in order to know the level of such species on human health and estimate their health effects. The analysis of the physical and chemical parameters of the groundwater in Qatar was conducted by Ahmed^33^, Manawi^34^ and Shomar^35,36^; however, their work did not include any investigation on the health effects associated with the elevated parameters. In other words, the study presented the existing levels without assessing the risks associated with consumption of groundwater for drinking and irrigation purposes. Hence, the novelty of the present work can be summarized by highlighting the main objective of the present work which is to give an overview of the chemical parameters of groundwater in Qatar as well as risk estimation associated with their consumption in irrigation knowing that groundwater might still be used in some parts of Qatar for irrigation without any pretreatment. To the best of our knowledge, there is no study reported on the assessment of the health risk correlated with the existing heavy metals levels in the groundwater of Qatar.

## Materials and methods

### Selection and collection of groundwater samples

**Figure 1:**
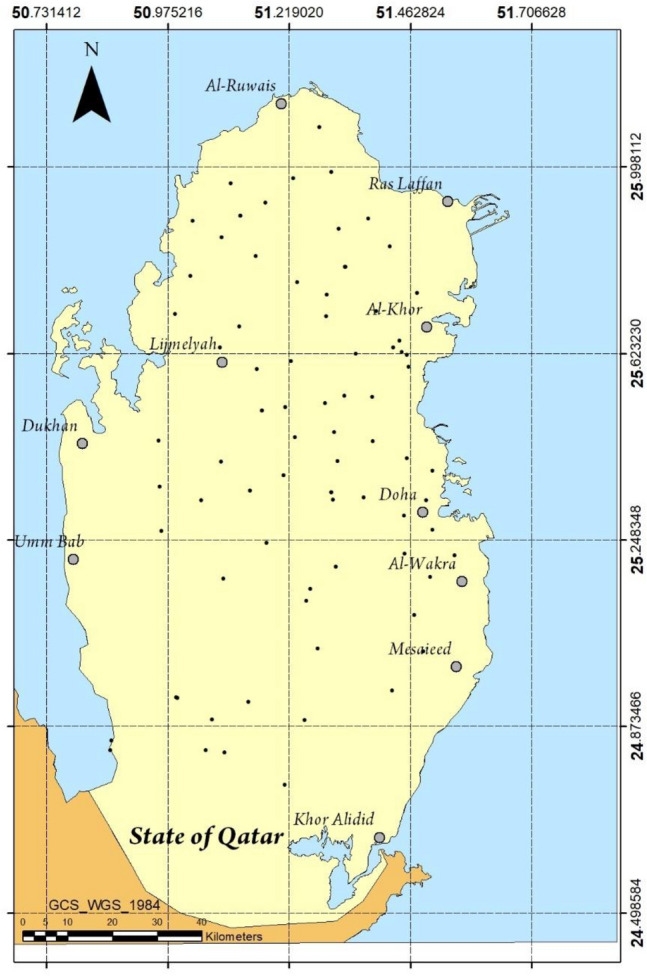
Location of groundwater sampling points selected in the present study.

In this study, 82 groundwater samples were collected from various parts around Qatar as shown in Fig. 1. The locations of the samples were carefully selected to cover the whole map of Qatar as well as give a representation of the various factors which may affect the quality of the collected samples such as groundwater well application, basin type, etc.

Prior to sample collection and in order to collect representative samples, the groundwater pump was turned on for 15 min in order to make sure that the collected samples are the well water and not the stagnant water kept in the pipes. Moreover, in the present work, for each groundwater well, three samples were collected and analyzed, and the average value was reported.

The limitation of the present study was in the selection of well distributed sampling points throughout the map of the country. Initially, we distributed the 82 groundwater samples were selected evenly to cover all the areas in Qatar. However, replacement sampling wells had to be selected in the vicinity of the sampling areas due to: (a) some of the selected wells dried up and the wells as well as the farms did not exist anymore and (b) some of the wells belong to industrial or private areas that did not want to participate in the study.

### Measurement of physical and chemical parameters

The physical measurements such as pH and TDS were evaluated in the field using HI-9829 multiparameter (Hanna Instruments, Woonsocket, Rhode Island, USA). The electrical conductivity (EC) was used to determine the total dissolved solids (TDS) in the collected groundwater samples using Eqs. (1) and (2). These equations were obtained from Schlumberger Water Services report that investigated the correlation between electric conductivity and total dissolved solids for Qatari groundwater between 1971 and 2009^7^.

1 $${\text{TDS }} = \, 0.{65 } \times {\text{ EC}}{-\!\!-}\left( {{\text{if EC }} < { 5}000 \, \upmu {\text{S}}/{\text{cm}}} \right)$$

2 $${\text{TDS }} = \, 0.{7}0 \, \times {\text{ EC}}{-\!\!-}\left( {{\text{if EC > 5}}000 \, \upmu {\text{S}}/{\text{cm}}} \right)$$

The analysis of major anions and cations in the samples [Potassium (K^+^), Sodium (Na^+^), Lithium (Li^+^), Magnesium (Mg^2+^), Calcium (Ca^2+^), Bromide (Br^−^), Fluoride (F^−^), Chloride (Cl^−^), Phosphate (PO_4_^3−^), Nitrite (NO_2_^−^), Nitrate (NO_3_^−^) and Sulfate (SO_4_^2−^)] were carried out using the ICS-5000+ Dionex Ion-Chromatography (Thermo Fisher Scientifics, Waltham, Massachusetts, USA). The columns used were separator column AS19 (2*250) mm as well as Guard Column type AG19(2*250) mm.

The alkalinity of the groundwater samples (in mg CaCO_3_/L) was determined using APHA 2320B method^37^ in which groundwater samples were titrated with sulfuric acid (with a normality of 0.1 N) in the presence of sodium carbonate (Na_2_CO_3_). Equation (3) was used to estimate the alkalinity^37^:

3 $$\text{Alkalinity }(\text{mg CaCO}_{3}/\text{L}) =\frac{A \times N \times \text{50,000}}{V}$$

where A is the volume of standard acid used which is sulfuric acid; N is the normality of sulfuric acid and V is the volume of the sample in ml.

The heavy metals analyzed in this work are: Lead (Pb), Iron (Fe), Copper (Cu), Nickel (Ni), Manganese (Mn), Zinc (Zn), Aluminum (Al), Mercury (Hg), Cadmium (Cd), Barium (Ba), Antimony (Sb), Arsenic (As), Chromium (Cr), Selenium (Se), Beryllium (Be) and Silver (Ag). The evaluation of the level of heavy metals in groundwater was carried out using 5800 ICP OES inductively coupled plasma–optical emission spectrometry (Agilent Technologies, Santa Clara, California, USA). The calibration curves were generated by dilution of the 1000 ppm Agilent Technologies standard stock calibration solution. The measurement of the parameters was repeated three times and the range as well as the mean values were included in this work. The mean values as well as the standard deviations were calculated using Microsoft Excel.

The correlation between the physicochemical parameters in the present work was analyzed by the determination of Pearson correlation coefficient between any two parameters. Equation (4) was used to estimate the Pearson correlation coefficient (r)^38^:

4 $$r= \frac{\sum ({x}_{i}-\overline{x })({y}_{i}-\overline{y })}{\sqrt{\sum ({x}_{i}-\overline{x }{)}^{2}\sum ({y}_{i}-\overline{y }{)}^{2}}}$$

where x_i_ and y_i_ represent the value of the x-variable and y-variables, respectively in any sample and $\overline{x }$ stands for mean value of the x-variable and $\overline{y }$ stands for the mean value of the y-variable.

### Development of Arc-GIS maps

The development of GIS maps was conducted using ArcGIS Desktop 10.8.1 software (version: 10.8.1.14362) which was developed by Environmental Systems Research Institute (https://www.esri.com) in which the map of Qatar was captured from Google Earth. The interpolation method followed in the present work was inverse distance weighted (IDW) in which the cell values were determined via a group of sample points that were weighted linearly. Moreover, the surface which is interpolated must be a variable that is location-dependent while the weight must be dependent or a function of inverse distance.

### Spatial analysis of pollution in groundwater

In the present work, the evaluation of pollution of groundwater by heavy metals was carried out using Nemerow comprehensive pollution index (NCPI). The main two parameters which are used to assess the pollution of any system with contaminants are pollution index (I_i_) and NCPI. I_i_ is used to express the pollution index of groundwater with a particular element i whereas NCPI is used to estimate the total pollution created by the presence of all the studied elements. Equations (1) and (2) were used to determine the pollution index (I_i_) and NCPI^39^:

5 $${I}_{i}= \frac{{C}_{i}}{{R}_{i}}$$

6 $$NCPI= \sqrt{\frac{{I}_{max}^{2}+ {I}_{avg}^{2}}{2}}$$

where C_i_ and R_i_ stand for the concentration and reference level of element i, respectively in mg/L. I_max_ and I_avg_ refer to the average and maximum value of the pollution index corresponding to element i. The classification of pollution with an element i according to the I_i_ value as well as the total pollution by heavy metals according to their NCPI value is listed in Table 1.

**Table 1:** Classification of pollution level in groundwater^39^.

I_i_/NCPI value	Pollution level
≤ 0.7	No pollution
0.7–1	Slight pollution
1–2	Light pollution
2–3	Moderate pollution
> 3	High pollution

### Evaluation of the non-carcinogenic human health risks

The estimation of the human health risk due to the exposure to heavy metals from water was covered by the United States Environmental Protection Agency (US EPA) reports in 1989^40^ and 2004^41^. The human health risk was attributed to take place as a result of 2 pathways which are the ingestion and dermal exposure. The total non-carcinogenic exposure can be figured out by estimating chronic daily intake (CDI) of heavy metals in mg/kg/day. The risk factor resulting from the exposure to non-carcinogenic ingestion and dermal doses can be obtained by determining the hazard quotients (HQ_ingestion_ and HQ_dermal_). Hazard Quotient is the ratio of the potential exposure level to a particular species to a reference dose level at which there will be no detrimental effects (RfD)^40^. The summation of the individual hazard quotients for various species and exposure pathways is called Hazard Index (HI).

The estimation of the health risks on human by heavy metals was conducted using Eqs. (7–11) which were obtained from US EPA reports^40,41^. The chronic daily intake (mg/kg/day) as well as the hazard quotients (HQ) received through ingestion and dermal exposures can be obtained using Eqs. (7–11)^41–44^:

7 $${CDI}_{ingestion}= \frac{C\times ED \times EF\times IR }{AT\times BW}$$

8 $${CDI}_{dermal}= \frac{C \times ED\times EF\times ET\times SA\times {K}_{p}\times {Ab}_{f}\times {C}_{f}}{AT \times BW}$$

9 $${HQ}_{ingestion}= \frac{{CDI}_{ingestion}}{RfD}$$

10 $${HQ}_{dermal}= \frac{{CDI}_{dermal}}{RfD}$$

11 $${\text{H}}{{\text{Q}}_{{\text{Total}}}}= {HQ}_{ingestion}+ {HQ}_{dermal}$$

where ED is the duration of exposure of 70 years; EF is the exposure frequency of 350 days; IR is the ingestion rate of drinking water of 2 L/day; AT is the average time for non-carcinogens of 25,550 days; BW is the average body weight of 70 kg; ET is the exposure time of 0.2 h/day; SA is the skin area that is exposed which is 18,000 cm^2^; Kp is the dermal permeability constant which has a value of 0.001 cm/h for Ba, B, Cu, Mn, Sr and V^41^ and 0.002 cm/h for Cr and 0.006 cm/h for Mo^41,44^; AB_f_ is the absorption factor corresponding to dermal exposure which is 0.001; C_f_ is the conversion factor between L and cm^3^ which accounts for 1 L = 1000 cm^3^; RfD is the reference dose for the investigated elements which is 70, 200, 3, 40, 20, 5, 1, 600 and 1 µg/kg/day for the oral ingestion pathway of Ba, B, Cr, Cu, Mn, Mo, Ag, Sr and V, respectively and 14, 180, 0.06, 12, 0.8, 1.9, 3, 120 and 0.01 µg/kg/day for the dermal pathway^44–46^. Hazard Index (HI) can be obtained by the summation of all of the individual hazard quotients due to the exposure to heavy metals through dermal and ingestion pathways.

### Evaluation of the carcinogenic human health risks

The potential of cancer risks due to the exposure of public to groundwater containing carcinogenic heavy metals can be estimated using the US EPA’s Incremental Lifetime Cancer Risk (ILCR) equation^42^:

12 $${\text{ILCR }} = {\text{ CSF }} \times {\text{ CDI}}$$

where CSF is the cancer slope factor (mg/kg/day) and CDI is the chronic daily intake of carcinogenic heavy metals through the lifetime consumption of groundwater (mg/kg/day).

The CSR can be determined from the risk generated when 1 mg of a particular contaminant is consumed per 1 kg of human body weight for a lifetime. In other words, the ILCR is used to show the probability of the potential health risks developed from the lifetime consumption of groundwater at the existing concentration of carcinogenic heavy metals. The ILCR value must be within the range of 1 × 10^–6^–1 × 10^–4^ in order to be considered acceptable by regulatory bodies.

## Results

### physical and chemical parameters of groundwater

**Figure 2:**
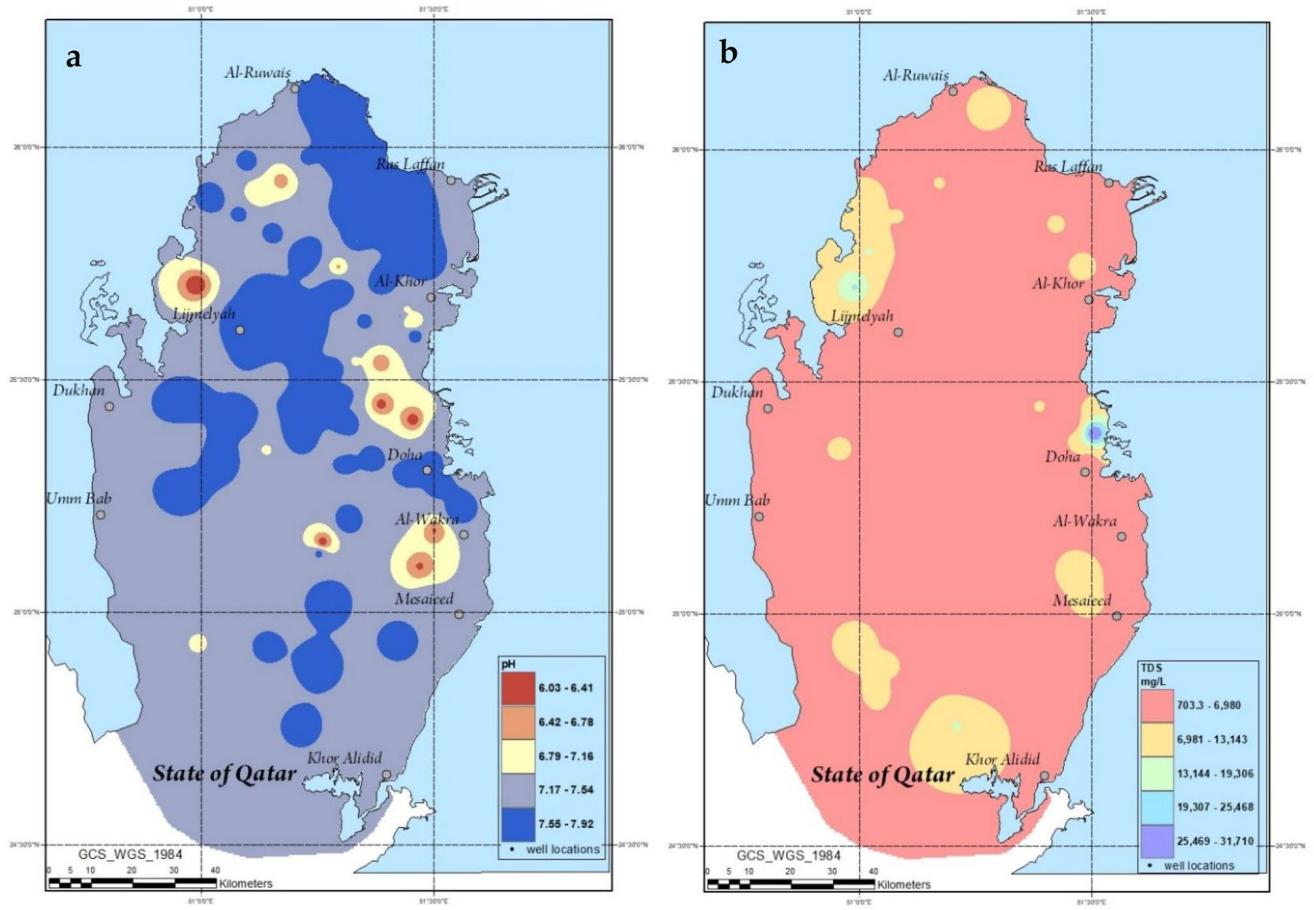
ArcGIS maps depicting the spatial variations in the level of pH (**a**) and TDS (**b**).

Figure S1 shows the investigated groundwater wells in Qatar. Table 2 shows the measured physical parameters of the investigated groundwater samples in comparison with WHO standards and Qatar Electricity and Water Corporation (Kahramaa) drinking water standards^47^. The local drinking water standards reported by Kahramaa, which owns and operates the distribution and transmission of electricity and water in Qatar, was issued in 2014. Figure 2 shows the variation in the pH, and TDS across the map of Qatar. As seen, the pH range and average of the studied samples was found to be 6.03 ± 0.235–7.92 ± 0.22 and 7.4 ± 0.48, respectively which was observed to fall within the WHO and Qatari drinking water standards. This was observed to be in good agreement with the work published by Shomar^36^.

**Table 2:** Physical parameters of the analyzed groundwater samples.

Physical parameter	Min	Max	Average	WHO standards	Kahramaa standards
pH	6.03 ± 0.235	7.92 ± 0.22	7.4 ± 0.48	6.5–8	6.5–8
EC (µS/cm)	1082 ± 51	45,300 ± 259	7885.4 ± 6641.7	500	150–500
TDS (mg/L)	703.3 ± 146	31,710 ± 715	5454 ± 4664	100	110–250

On the other hand, the TDS values had a range of 703.3 ± 146–31,710 ± 715 mg/L. The United States Salinity Laboratory (USSL) issued a handbook on the categorization of irrigation water^48,49^. Table 3 depicts the categorization of irrigation water according to their EC and TDS. As seen, the salinity level in the analyzed groundwater was found to be high. In fact, about 5% of the samples had salinity levels between 500 and 1500 mg/L which was reported by USSL to damage plants with low salinity tolerance. On the other hand, about 95% of the samples were found to have a hazard class that is very high with consequent damage to high salinity tolerant plants. Moreover, the average TDS value was 5454 ± 4664 mg/L which is way greater than 1500 mg/L limit which describes water with very high salinity.

**Table 3:** Categorization of saline water according to their EC and TDS as reported by USSL^48,49^.

Hazard class	EC (µS/cm)	TDS (mg/L)	Potential injury
Low	< 250	< 150	Salinity hazard
Medium	250–750	150–500	May damage plants with salt sensitivity
High	750–2250	500–1500	Will damage plants with low salinity tolerance
Very high	> 2250	> 1500	Damage to high salinity tolerance plants

The spatial distribution analysis of TDS in Qatar conducted by Shomar showed that the northern parts of Qatar are characterized with more brackish groundwater compared with south^35^. This can be attributed to the fact that up to 10% of the estimated annual rainfall of 25 million cubic meters in Qatar occurs on the northern part whereas up to 5% only takes place on the southern parts^50^.

**Figure 3:**
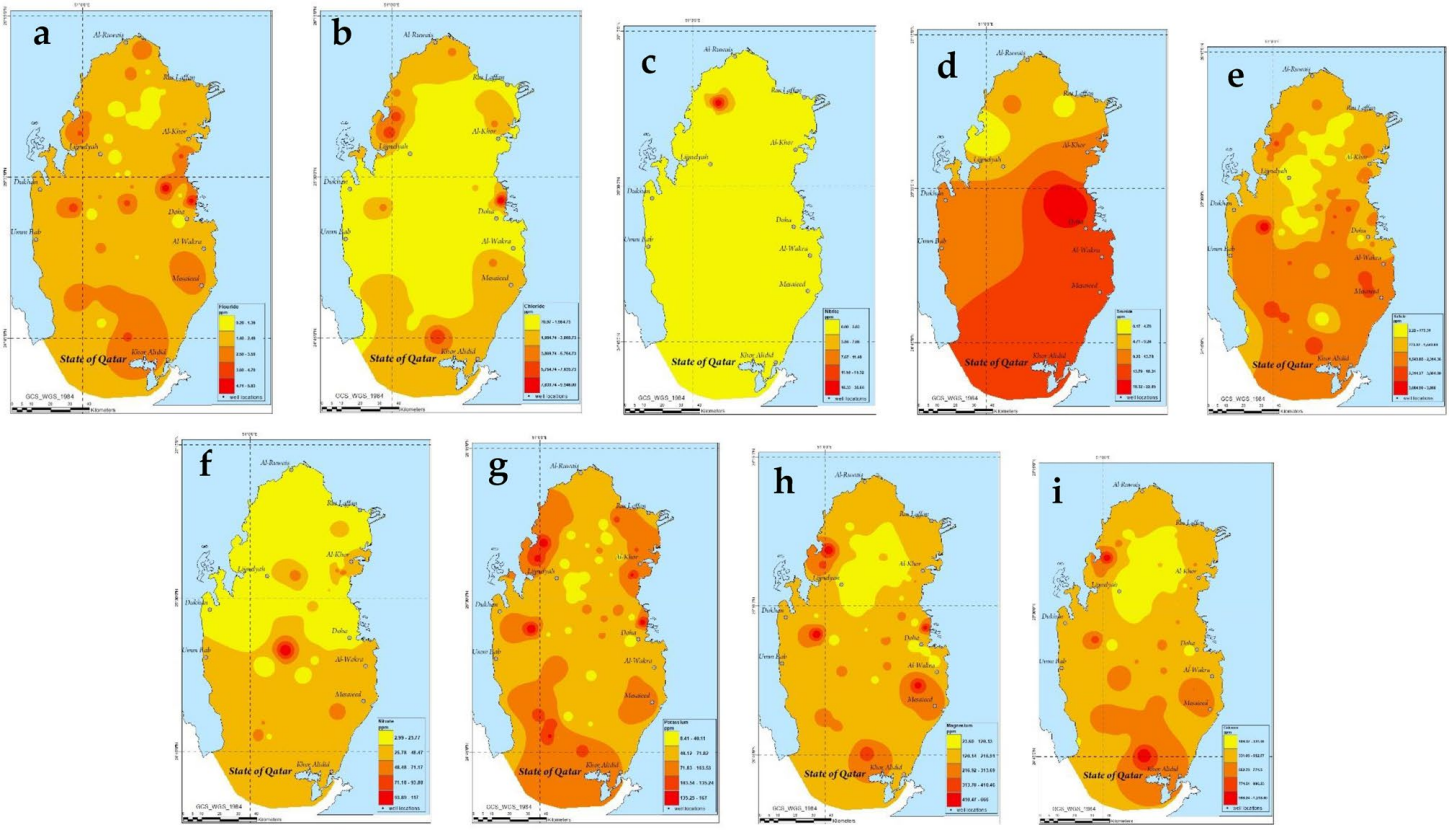
ArcGIS maps depicting the spatial variations in the level of fluoride (**a**), chloride (**b**), nitrite (**c**), bromide (**d**), sulfate (**e**), nitrate (**f**), potassium (**g**), magnesium (**h**) and calcium (**i**).

In the present work, the determination of the major cations and anions was carried using ion chromatography. Table 4 shows the level of Potassium (K^+^), Sodium (Na^+^), Lithium (Li^+^), Magnesium (Mg^2+^), Calcium (Ca^2+^), Bromide (Br^−^), Fluoride (F^−^), Chloride (Cl^−^), Phosphate (PO_4_^3−^), Nitrite (NO_2_^−^), Nitrate (NO_3_^−^) and Sulfate (SO_4_^2−^) in 82 groundwater samples in Qatar. Moreover, Fig. 3 shows ArcGIS maps depicting the spatial variations in the level of major cations and anions in Qatar. The level of chloride, bromide, sulphate, sodium, magnesium and calcium in the present study were found to exceed Qatar drinking and crop irrigation standards as presented in Table 4. The sulphate level in the groundwater ranged between 2.22 and 3680 mg/L with a mean of 1383 ± 901.34 mg/L. While the recommended sulphate level in drinking water should not exceed 50 mg/L, it was observed that 76 locations (or 93% of the studied samples) had sulphate levels above that. Similarly, the Qatari crop irrigation limit for sulphate should be below 400 mg/L^7^. It was found that 67 locations (82% of the studied samples) exceeded the Qatari crop irrigation limit. The elevation in the level of sodium and chloride in the studied samples above the drinking standards showed 78 and 81 exceedances, respectively. Likewise, the elevation in the level of calcium in the studied samples above the drinking standards of 80 mg/L showed 82 exceedances or 100% of the analyzed samples. It was worth pointing out that the bromide level in 7 locations in the present study exceeded Kahramaa’s drinking water limit of 0.1 mg/L. Boron (B) is a trace element that leaches out from natural rocks due to its well solubility in water and can be found in some water bodies such as groundwater and seawater. Boron is key for osteogenesis and bone development; however, boron can be harmful to humans above safe levels. The Qatari drinking water guidelines for boron should not exceed 1 mg/L. Boron level as high as 4.1 mg/L was observed in the present study which was greater than the allowable limit set by WHO of 2.4 mg/L and Kahramaa of 1 mg/L in drinking water. About 29% of the studied groundwater samples showed boron levels that are greater than the WHO drinking water limit of 2.4 mg/L^51^ whereas 80% showed boron levels that exceeded Kahramaa drinking water standards. Boron at high levels can cause toxicity to plants and may be responsible for several male fertility problems. The presence of boron in groundwater could be attributed to the desorption of boron from mineral rocks by infiltration of rainwater as well as the infiltration of wastewater which is characterized with elevated boron levels from detergents and soaps^33,52^. The boron level in groundwater was found to agree with the level reported by Ahmad^33^ with a range of 0.3–3.8 mg/L and a mean of 1.8 mg/L. Moreover, it was also found to agree well with the boron level reported by Schlumberger during their analysis of groundwater in Qatar with a range of 0.25–5.63 mg/L and an average of 2.06 mg/L^7^. The removal of boron and other major contaminants can be achieved by using various treatment techniques such as membrane filtration or adsorption^53–65^. For instance, the preparation of novel sustainable adsorbents from natural materials, such as banana peel^62^, olive stones^63^ and pistachio shell^65^ as well as carbon nanotubes^64^ for the removal of various pollutants in water was covered in literature. The developed adsorbents such as activated carbon and biochar performed well in the removal of PAH as well as pharmaceuticals (ciprofloxacin and diclofenac) from wastewater. The studies reported that the adsorbent can be potentially used for large scale application^65^.

**Table 4:** The major anions as well as cations analyzed in the collected groundwater samples compared with Kahramaa and WHO standards.

Ion	Present work			Kahramaa^47^			WHO (drinking water)
	Min	Max	Avg	Min	Max	Min	Max
Fluoride (mg/L)	0.28	5.83	2.14 ± 1.06	–	1.5	1.5	10
Chloride (mg/L)	70.97	9,946	1865 ± 2123	–	< 80	–	250
Nitrite (mg/L)	0	35.56	0.46	–	0.1	–	50
Bromide (mg/L)	0.17	22.85	11.01 ± 7.07	–	< 0.1	–	0.5
Sulfate (mg/L)	2.22	3860	1383 ± 895	–	50	–	–
Nitrate (mg/L)	2.99	117	27.07 ± 24.10	–	10	–	–
Phosphate (mg/L)	0	0.427	0 ± 0.05	–	0.01	–	
Lithium (mg/L)	0.1185	5.01	1.07 ± 1.28	–	0.05	–	
Sodium (mg/L)	17.2	14,873	1554.71 ± 2244.51	–	80	–	200
Potassium (mg/L)	1.9	665.4	106.6 ± 101.1	–	4	–	
Magnesium (mg/L)	41.078	1024.98	203.881 ± 157.44	–	30	–	150
Calcium (mg/L)	88.28	1218	438.15 ± 232.04	–	80	–	200
Boron (B)	0.05	4.151	1.95 ± 0.97	–	1	–	2.4

**Figure 4:**
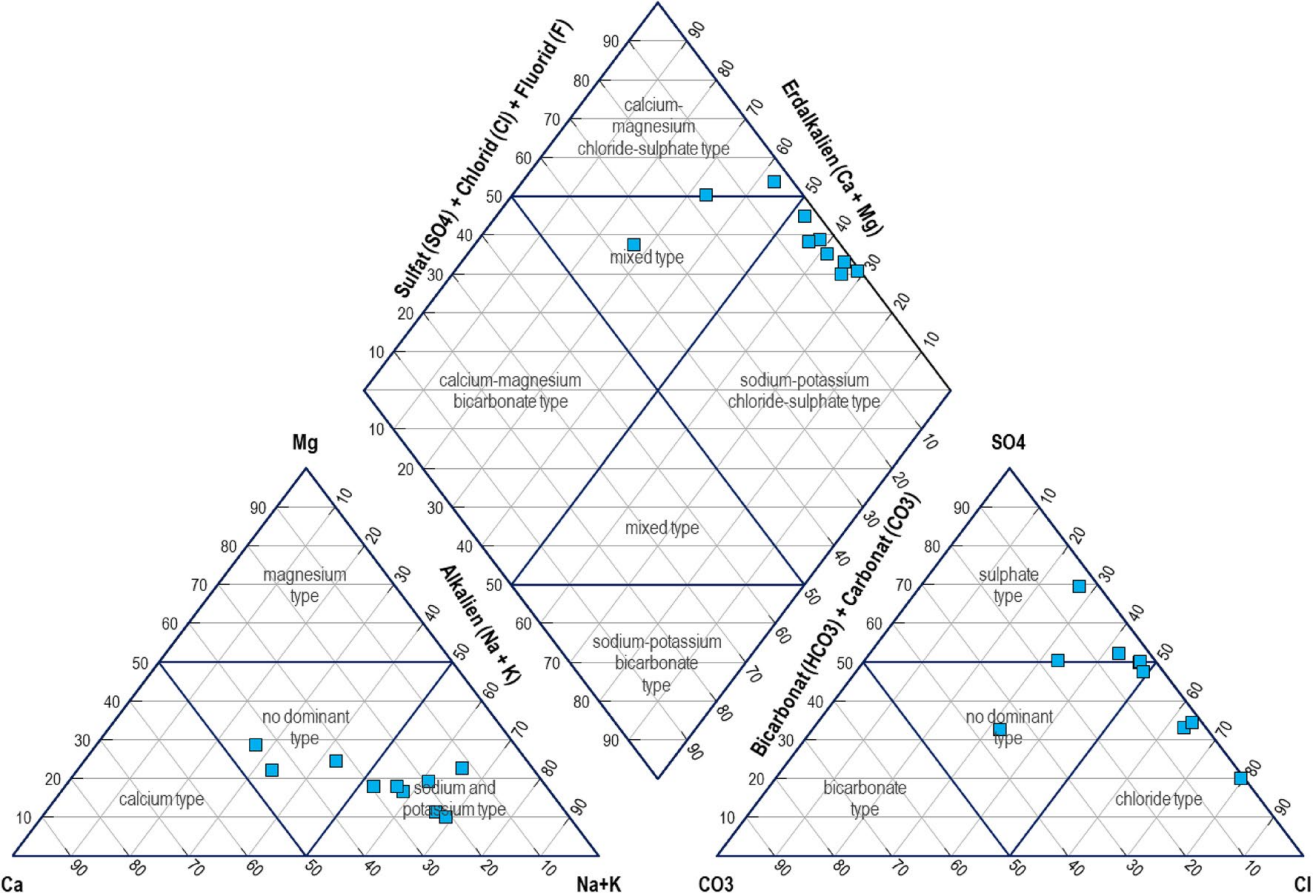
Piper diagram for groundwater in Qatar.

The piper diagram plotted for 10 groundwater samples is shown in Fig. 4. The cations triangle in the present work was observed to be dominated with sodium and potassium type while the anions triangle was dominated by chloride and sulfate types. The plotting of the investigated groundwater samples on the diamond-shaped diagram in Fig. 4 showed that the groundwater in Qatar is dominated by sodium–potassium-chloride-sulfate as well as calcium-magnesium-chloride-sulfate types. In other words, groundwater in Qatar was found to mainly exist in 2 areas which are: (1) Ca-Mg and Cl-SO_4_ as well as (2) Na–K and Cl-SO_4_. This was observed to be in good agreement with the characterization and development of piper diagrams for groundwater in Qatar^7,33^ which reported the groundwater to vary from sodium to calcium type and chloride to sulphate type. It was also reported that most of the groundwater in Qatar is dominated by sulfate and chloride anions.

The spatial distribution analysis of the major cations and anions in Qatar depicted in Fig. 3 shows that northern parts have relatively lower concentrations of these elements. This could be confirmed by observing the levels of Fluoride, Chloride, Nitrite, Bromide, Sulfate, Nitrate, Potassium, Magnesium, Calcium and Boron which are concentrated in the southern part of the country. This can be attributed to the fact that up to 10% of the estimated annual rainfall of 25 million cubic meters in Qatar occurs on the northern part whereas up to 5% only takes place on the southern parts^50^.

**Figure 5:**
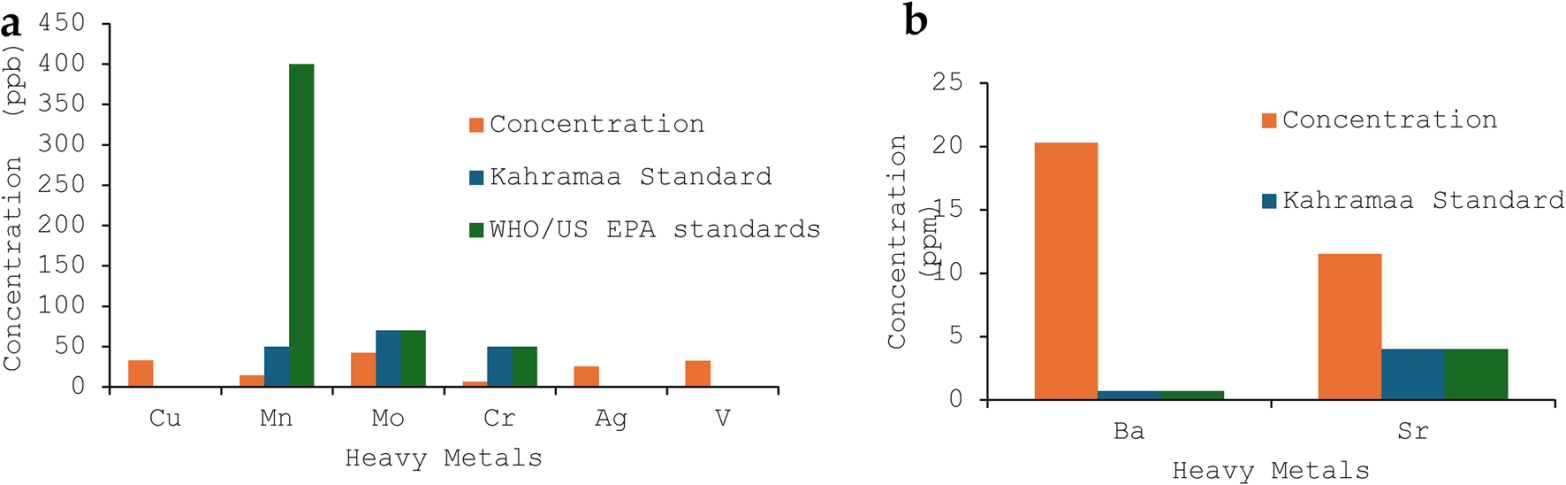
The average level of heavy metals in the groundwater samples compared with Qatari, WHO or US EPA standards in ppb (**a**) and ppm (**b**). *LOR: limit of reporting, which is 0.05 ppb for Hg, 1 ppb for Pb, 0.5 ppb for Mn, 1 ppb for Ni, 1 ppb for Cu, 0.05 ppm for Fe, 0.05 ppm for Zn, 0.05 ppm for Al, 0.5 ppb for Cd, 0.1 ppm for Sb, 0.05 ppm for As, 0.1 ppm for Se, 0.5 ppb for Be, 1 ppb for Ag, 0.1 ppm for TI, and 0.1 ppm for U.

**Figure 6:**
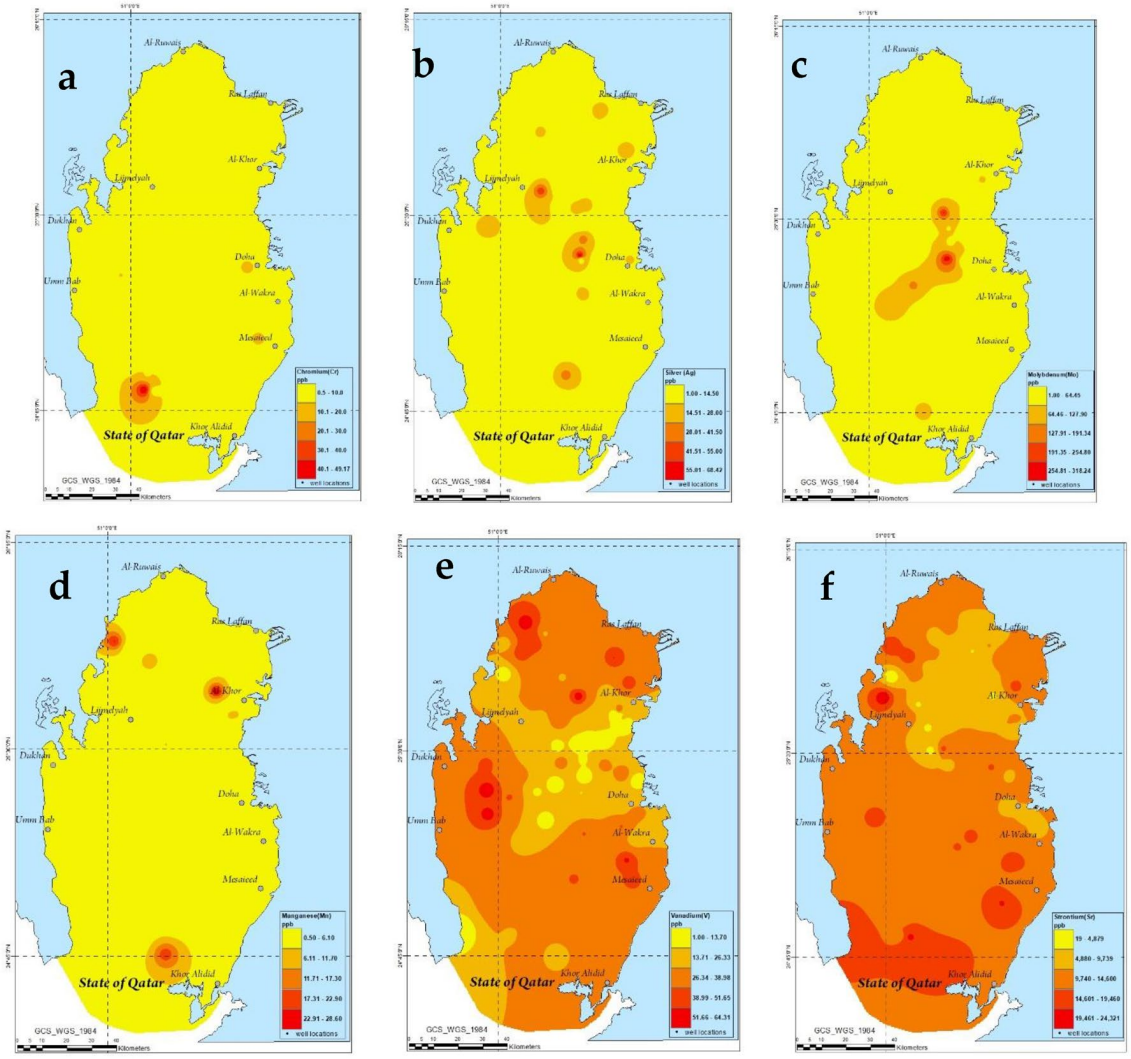
ArcGIS maps depicting the spatial variations in the level of chromium (**a**), silver (**b**), molybdenum (**c**), manganese (**d**), vanadium (**e**) and strontium (**f**).

The measurement of the heavy metals content in the groundwater was conducted using ICP-OES. Figure 5 depicts the level of some of the heavy metals in the groundwater compared with Qatari, WHO or US EPA standards in ppb (a) and ppm (b). The determination of the levels of heavy metals in the present work showed that all the sample results fall within the acceptable limits set by WHO and Qatari standards except for molybdenum and strontium which were found to be higher than the WHO and Qatari Standards. Moreover, Fig. 6 depicts the spatial variations in the level of heavy metals in groundwater of Qatar using ArcGIS maps. In the present work, with the exception of few outliers, the elevation in the heavy metals levels in the groundwater in the present study do not indicate the existence of severe health threat^7^. The main concern associated with groundwater in Qatar is related to the presence of sodium and high salinity levels.

The level of Mo in the present study was observed to have a range of 1–319.5 ppb and an average of 37.9 ppb which was found to agree with the level reported by Ahmad (Range: 7.8–294 ppb and average: 53.9 ppb)^33^. Moreover, the analysis reported by Schlumberger in 2009 showed similar levels and attributed the elevation in molybdenum due to the presence of evaporite conditions (high temperature accompanied with low precipitation rate) in Qatar^7^. The spatial distribution analysis of the elevation in molybdenum level showed their occurrence in the center areas of Qatar. The reason behind the elevation in the level of molybdenum could be attributed to the enrichment of these metals by the existence of hydrogeochemical environment. This also could be attributed to the contamination from the well itself as a result of the corrosion in the pump or well casing^7^. It is worth pointing out that the elevation in the molybdenum level in the groundwater was few orders of magnitude higher than normal levels which indicates the anthropogenic sources of this elevation^7^. Moreover, Kuiper investigated the level of Mo in groundwater of Qatar^66^ and reported a range of 2.7–103 ppb and average: 26.9 ppb^35^. The reason behind the elevation in the Mo level in groundwater can be attributed to several factors such as (1) longer groundwater residence time, (2) abundance of sulfide and iron oxides minerals, (3) neutral to alkaline groundwater pH and (4) existence of reducing conditions in the aquifer^66,67^. The presence of saline groundwater was reported to enrich Mo levels in groundwater as a result of the impediment in the sorption of Mo in soils that are characterized with arid and alkaline conditions^66,68–71^. As seen in the present work, the geological and chemical properties of the groundwater in Qatar was observed to agree with the factors discussed above in terms of soil salinity, alkalinity and pH. The existence of dolomite, gypsum and limestone formations in Qatar will give rise to the elevation in Mo level following the demineralization of geological formations.

The level of lead in the groundwater of Qatar was observed to be below the detection level of 1 µg/L which is definitely below the WHO drinking water standard of 10 µg/L. This was found to agree with the work published by Ahmad^33^ which reported the lead level in the groundwater of Qatar to be below the detection level.

The level of strontium in the present work had a range of 16.39–24,347.58 and an average of 11,516.73 ± 4802.95 µg/L. The reported levels are not considered high as there are no WHO guidelines/limit on the allowable level of strontium in drinking water. A recommended level of 4 mg/L^7,72^, followed in the present study, was reported by US EPA and was also adopted by the local distributor of water in Qatar^73^. Moreover, the range of the reported levels were found to agree with the levels found in Ahmad’s work who investigated the levels of Sr in groundwater of Qatar and reported similar levels. For instance, the Sr level in groundwater of Qatar was reported to have a range of 3534–20,273 and an average of 13,226 µg/L^33^. The Sr level in groundwater was attributed by Ahmad to occur due to the evaporite deposits^33^. Moreover, the level of Sr in groundwater of Qatar was reported by Shomar^35^ who analyzed the level of Sr in groundwater as well as soil and reported a ratio of Ca:Sr in soil to be correlated with the ratio in groundwater. The increase in the salinity of the soil was found to increase the level of Sr in soil due to the characteristic high level of Cl^−^ and SO_4_ in saline soil which is also rich in Sr^35^.

Table 5 shows the Pearson correlation coefficients between the investigated parameters in the present work. Normally, Pearson correlation coefficient values vary between − 1 and 1. A Pearson correlation coefficient of − 1 indicates a completely negative linear correlation between the two variables whereas a value of 1 indicates a completely positive correlation. Moreover, a value of 0 designates a no correlation between the investigated variables^74^. Furthermore, a Pearson correlation coefficient < 0.3 designates a weak correlation whereas values between 0.3 and 0.7 have moderate correlations and correlations having values ≥ 0.7 are considered strongly correlated^75^. As seen in Table 5, pH showed an intermediate correlation with F^−^ with a value of − 0.32 which indicates that pH and F^−^ are inversely proportional to each other. An inverse relationship between pH and F^−^ was reported by Sivasankar^76^ and Umarani^77^ in Tamilnadu (India). Moreover, the correlation coefficients between TDS and Cl^−^ showed a strong correlation with a Pearson correlation coefficient of 0.94. On the other hand, TDS was moderately correlated with F^−^, SO_4_^2−^, NO_3_^−^ and Ca^2+^, Na^+^, Mg^2+^ and K^+^ with a correlation coefficient range of 0.32–0.64.This was found to agree with the values reported by Sakram^78^ who reported a moderate correlation between the TDS and F^−^, Ca^2+^, Na^+^, Mg^2+^ and K^+^. Furthermore, Ca^2+^ showed a strong correlation with Cl^−^ and SO_4_^2−^ with a correlation coefficient of 0.71 and 0.82, respectively. Likewise, Mg^2+^ and Na^+^ were strongly correlated with a correlation coefficient of 0.90.

**Table 5:** Pearson correlation coefficients between the investigated parameters.

	pH	TDS	Cl^−^	F^−^	SO_4_^2−^	NO_3_^−^	Na^+^	K^+^	Mg^2+^	Ca^2+^	Ag	Mn	Cr	Mo	Sr	V
pH	1.00	− 0.27	− 0.21	− 0.32	− 0.24	0.01	− 0.10	− 0.11	− 0.14	− 0.20	0.17	0.09	0.11	− 0.04	− 0.22	0.01
TDS		1.00	0.94	0.61	0.54	0.32	0.64	0.63	0.64	0.61	− 0.16	0.04	0.06	0.07	0.45	− 0.02
Cl^−^			1.00	0.59	0.58	0.26	0.56	0.54	0.58	0.71	− 0.17	0.14	0.05	0.03	0.43	0.03
F^−^				1.00	0.52	0.16	0.34	0.38	0.43	0.58	− 0.21	0.02	− 0.17	0.10	0.36	− 0.06
SO_4_^2−^					1.00	0.47	0.23	0.28	0.39	0.82	− 0.19	− 0.03	0.16	0.33	0.42	0.13
NO_3_^−^						1.00	0.35	0.34	0.35	0.35	− 0.05	0.04	0.16	0.27	0.22	− 0.26
Na^+^							1.00	0.97	0.90	0.19	− 0.14	0.11	0.04	0.06	0.45	− 0.13
K^+^								1.00	0.91	0.21	− 0.14	0.09	0.03	0.12	0.50	− 0.12
Mg^2+^									1.00	0.39	− 0.16	0.05	− 0.02	0.15	0.58	− 0.12
Ca^2+^										1.00	− 0.16	0.08	0.00	0.25	0.44	0.06
Ag											1.00	− 0.08	− 0.03	0.05	− 0.14	− 0.16
Mn												1.00	− 0.10	− 0.03	0.04	− 0.15
Cr													1.00	− 0.05	0.06	0.13
Mo														1.00	0.18	− 0.23
Sr															1.00	0.09
V																1.00

On the other hand, Sr has also a moderate correlation with TDS, Na^+^, Cl^−^, F^−^, SO_4_^2−^, Mg^2+^, Ca^2+^ and K^+^ as confirmed by the Pearson correlation coefficients which were between 0.3 and 0.7. These correlations were also confirmed by Ahmad ^33^ who reported the Pearson correlation coefficients of Sr with TDS, Na^+^, K^+^, Ca^2+^ Mg^2+^, F^−^, Cl^−^ and SO_4_^2−^ in groundwater of Qatar to be 0.58, 0.46, 0.45, 0.65 and 0.68, 0.59, 0.49 and 0.67 respectively, which were found to be in the moderately-correlated zone. The level of NO_3_^−^ had a intermediate correlation with SO_4_^2−^ with a Pearson correlation coefficient of 0.47. This was found to agree with the correlation reported by Burhan^79^ with a Pearson correlation coefficient < 0.7.

On the other hand, the correlation between Mo and SO_4_^2−^ in the present work was observed to be moderately correlated with a Pearson correlation coefficient of 0.33. This was also reported by Tasneem who analyzed the hydrochemistry of the groundwater in Wadi Al Arab aquifer, which is located on the northwestern side of Jordan^80^ and found a moderate correlation between Mo and SO_4_^2−^ in groundwater.

### Pollution assessment

**Figure 7:**
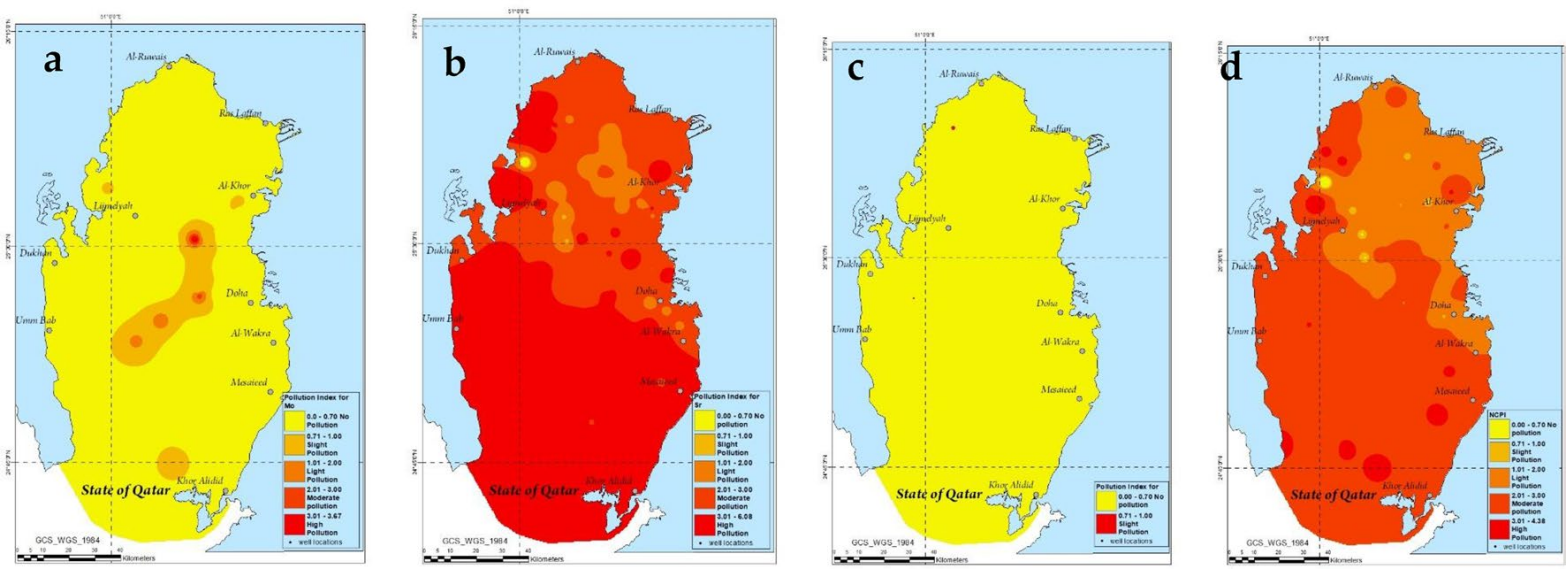
ArcGIS maps depicting the spatial variations in the pollution index of molybdenum (**a**), strontium (**b**) and vanadium (**c**) as well as NCPI of the heavy metals (**d**) in groundwater.

Table 6 shows the statistical analysis of the pollution index of as well as NCPI for the investigated 82 groundwater samples. The estimation of the pollution index and NCPI helps categorize the degree of pollution in the groundwater by comparing the I_i_ and NCPI values with the values published in US EPA’s report. The average I_i_ values in the present work were found to range from 0.04 to 2.84 whereas the maximum values ranged between 0.58 and 6.09. According to the average I_i_ values in the table, degree of pollution in the groundwater was observed to increase in this order: Mn < Cr < Ag < V < Mo < Sr. As per the classification of pollution in Table 1, there is no pollution in terms of Mn, Ag, Cr, V and Mo; however, the average pollution index value shows that there is a moderate pollution in terms of the Sr content in the groundwater. The level of Sr in groundwater was found to be higher than the US EPA’s recommended level in drinking water of 4 mg/L despite the absence of any recommended Sr level in WHO’s drinking water guidelines. The estimation of the pollution index in the present work was based on the US EPA’s recommended level in drinking water (4 mg/L)^7,72^. This was also seen in the pollution index values corresponding to the average level as well as the maximum level of Sr which were 2.84 and 6.09, respectively. According to Table 6, the pollution indices corresponding to the average and maximum levels of Sr categorize the groundwater in Qatar into moderate and high pollution, respectively. Also, in terms of Sr level, about 19% of the samples showed light pollution and 28% showed moderate pollution whereas 46% showed high pollution. In terms of Vanadium, despite showing a no pollution category based on the average I_i_ value, the maximum Ii value corresponding to Vanadium (Ii = 0.74) was found to be in the slight pollution category based on a safe limit of 100 µg/L suggested by Dr. Meisch^46,81^. Moreover, despite the fact that the average Mo level in groundwater fall in the no pollution category, the highest level of Mo in groundwater showed a pollution index of 4.56 which falls in the high pollution category based on a US-EPA level of 70 µg/L^82^. About 14% of the samples have either slight or light pollution while 5% of the samples showed moderate or high Mo pollution. About 82% of the samples showed no Mo pollution. The spatial variations of the level of Mo and Sr in Qatar can be depicted in Arc GIS maps in Fig. 7.

**Table 6:** Pollution index and NCPI of heavy metals in groundwater.

	Pollution Index (I_i_)						NCPI
	Ag	Cr	Mn	Mo	Sr	V	
Avg	0.07	0.05	0.04	0.54	2.84	0.27	2.08
Min	0.01	0.00	0.00	0.00	0.00	0.00	0.01
Max	0.69	0.99	0.58	4.56	6.09	0.74	4.39
SD	0.13	0.12	0.10	0.72	1.23	0.17	0.90

The Nemerow Index values in the present work was found to range from 0.01 to 4.39 with an average of 2.08. The average NCPI value categorizes the pollution in groundwater into moderate pollution with the main pollution source coming from Sr with a pollution index of 2.88. About 7% of the samples showed an NCPI in the slight pollution category whereas 30% were in the light pollution category. About 42% of the samples showed NCPI in moderate pollution and 16% in high pollution. Figure 7 shows the spatial variation in the level of NCPI across the map of Qatar.

The main findings of this work were observed to agree with the work reported in literature. For example, the evaluation of the heavy metal pollution in Huaibei Plain farmlands (Xiaoxian County) in China was performed by Zhang^28^. The study analyzed the levels of Ni, Cr, Cd, Cu and Zn in 69 groundwater samples and health impacts on public. The work showed that the NCPI had a range of 0.25–9.3 with an average of 1.9. Moreover, the assessment of the health effects associated with Pb, As and Cr^6+^ in groundwater in the Jinghui Canal in China was reported by Zhang^23^. The study showed that 31.9% of the samples had an NCPI range of 1–4.2 while 43% had NCPI values > 4.2. It was also reported that there is a serious pollution in the Jinghui canal, and the groundwater is not suitable for drinking or irrigation. Similarly, Mamat^29^ also analyzed the pollution of Ibinur Lake Basin in China with Pb, Se, As, Mn, Zn, Cu, Cd and Cr by analyzing 75 groundwater samples. The study revealed that the NCPI of the studied groundwater samples ranged between 0.1 and 3. Likewise, Singh^27^ studied the pollution of groundwater in Haridwar district in India and found out that 84% of the samples were slightly polluted while the pollution index had a range of 0.99–4.32. The main findings of the present work in comparison with the results reported in literature is presented in Table 7.

**Table 7:** The reported studies on health risk assessment of trace metals in groundwater from literature.

Country	GW well application	Trace metals	Reported risk assessments (PI, NCPI, HI, ILCR)	Ref
China	Irrigation	Ni, Cr, Cd, Cu and Zn	NCPI: 0.25–9.3 19 samples showed ILCR of Cr > 10^–4^ 27.5% of samples had human health risks	^28^
China	Irrigation	Pb, As and Cr^6+^	31.9% of the samples had an NCPI range of 1–3.96 while 43% had NCPI values > 4.2 Serious pollution in the Jinghui canal as the GW is not suitable for drinking or irrigation	^23^
India	Irrigation		NCPI: 0.99–4.32	^27^
China	Irrigation	Pb, Se, As, Mn, Zn, Cu, Cd and Cr	NCPI: 0.1–3	^29^
Iran	Drinking	Cd, Mo, Pb, Zn, Ba, Cr, Ni and Cu	CDI_Total_: 3.3 × 10^–5^–5.4 × 10^–3^ HI: 5.3 × 10^–7^–8.05 × 10^–3^ ILCR: 2.9 × 10^–5^–1.5 × 10^–2^ Cr is the major contributor to cancer	^42^
China	Drinking and Irrigation	Al, Mn Hg Ni As, Cr, Cu, Cd, Co, and Zn	Al level exceeded the cleaning level Al contributed the most to GW pollution with an average of 65.7%	^30^
Nigeria	Drinking	Mn, Cu, Cd Pb, Cr and Ni	ILCR: 48.5 CDI_max_ of Pb: 0.41 HQ_max_ of Pb: 1030	^32^
China	Irrigation	Ni, Se, B, Mn, Al and Zn	CDI: 5.1 × 10^–5^–6.9 × 10^–4^ HQ_Total_: 5.9 1 × 10^–3^–7.8 1 × 10^–1^ Zn generated the highest non-carcinogenic human hazard	^45^
China	Irrigation	Zn, Ni, Cr, Cd, Mn and Cu	PI: 0.01–3 NCPI: 0.23–2.25 CDI: 3.5 × 10^–4^–1.9 × 10^–3^ mg/kg/day HQ_Total_: 6.1 × 10^–3^–4.9 × 10^–1^ (adults) and 7 × 10^–3^–6.7 × 10^–1^ (for children) HI: 0.78–1.05 Cd was responsible for the highest health risks	^44^
Qatar	Irrigation	Mn, Cr, Ag, V, Mo and Sr	PI: 0–6.09 NCPI: 0–4.39 ILCR: 5.6 × 10^–4^–5.5 × 10^–2^ CDI: 1.4 × 10^–5^–6.7 × 10^–1^ mg/kg/day HI: 0–3.2	Present work

### Evaluation of the non-carcinogenic human health risk

The estimation of the non-carcinogenic human risk to human health was performed using Eqs. (7–11). In the present work, the chronic daily intake (CDI) as well as the hazard quotient and hazard indices for the investigated heavy metals (Ag, Mn, Cr, V, Mo and Sr) in all of the studied groundwater samples was calculated. Moreover, Table 8 lists the min, max as well as the average values corresponding to CDI_Total_ and HQ. It was observed that the total chronic daily intake (through ingestion and dermal pathways) for all the studied metals was found to have a range of 1.4 × 10^–5^–6.7 × 10^–1^ mg/kg/day. The chronic daily intake through ingestion pathway was observed to be more pronounced in comparison with the dermal pathway. For instance, the CDI_ingestion_ is more than 500,000 times the CDI_dermal_ of Sr and V and more than 270,000 the value of CDI_dermal_ corresponding to Cr. This can be attributed to the low absorption rate at the skin compared to the high absorption rate through the gastrointestinal tract or digestive system which increases the toxicity of ingested substances. The protection mechanism accompanied by the smaller surface area of the skin will also be responsible for the reduction of the CDI_dermal_ compared with CDI_ingestion_ and hence, ingestion is the major pathway as confirmed by the CDI_ingestion_ and CDI_dermal_ values in the present work. According to the minimum values of the total chronic daily intake of heavy metals, CDI_Total_ was found to range between 1.4 × 10^–5^ and 4.5 × 10^–4^ and increase in this order: Cr = Mn < Mo = V = Ag < Sr. Furthermore, according to the maximum values of the total chronic daily intake of heavy metals, CDI_Total_ had a range of 7.9 × 10^–4^–6.7 × 10^–1^ mg/kg/day and were observed to increase in this order: Mn < Cr < Ag < V < Mo < Sr. The maximum CDI_Total_ value encountered corresponded to Sr which had a value of 6.7 × 10^–1^ mg/kg/day. According to the average values, it was found that total chronic daily intake of heavy metals followed this order: Mn < Cr < Ag < V < Mo < Sr. Moreover, the average CDI_Total_ had ranged between 4.9 × 10^–5^ and 3.1 × 10^–1^ mg/kg/day.

**Table 8:** Summary of the CDI_Total_ as well as HQ for all of the studied heavy metals.

Element	CDI_Total_			HQ		
	Min	Max	Average	Min	Max	Average
Cr	1.37E−05	1.35E−03	7.04E−05	4.57E−03	4.51E−01	2.35E−02
Mn	1.37E−05	7.89E−04	4.98E−05	6.85E−04	3.95E−02	2.49E−03
Mo	2.74E−05	8.75E−03	1.03E−03	5.48E−03	1.75E+00	2.06E−01
Ag	2.74E−05	1.89E−03	1.90E−04	2.74E−02	1.89E+00	1.90E−01
Sr	4.49E−04	6.67E−01	3.12E−01	7.49E−04	1.11E+00	5.20E−01
V	2.74E−05	2.02E−03	7.41E−04	2.74E−02	4.51E−01	7.41E−01

The average hazard quotients of all the studied heavy metals presented in Table 8 were found to range between 2.5 × 10^–3^ and 7.4 × 10^–1^ and increase in this order: Mn < Cr < Ag < Mo < Sr < V. On the other hand, the maximum HQ ranged between 3.9 × 10^–2^ and 1.89 and followed the order: Mn < Cr = V < Sr < Mo < Ag. The total hazard quotient value at the minimum trace metals levels was observed to range between 7.5 × 10^–4^ and 2.7 × 10^–2^. Likewise, the total hazard quotient at the maximum level of trace metals had a range of 3.9 × 10^–2^ and 1.75. The order of the HQ_Total_ values at the minimum levels was Mn < Sr < Cr < Mo < Ag = V while the order at the maximum levels was Mn < Cr = V < Sr < Mo < Ag. Despite the absence of any mention of vanadium metal in Kahramaa, WHO or US EPA drinking water standards, the evaluation of the non-carcinogenic human health risk associated with vanadium was based on the reference dose (RfD) for ingestion and dermal exposure recommended by US EPA and reported in literature^44–46^. The reference doses for oral ingestion (1 µg/kg/day) and dermal exposure (0.01 µg/kg/day) were used in estimating non-carcinogenic human health risk using Eqs. (9 and 10). For instance, the estimation of the health hazards in associated with vanadium in the groundwater water in lower Yellow River in China at a maximum vanadium level of 58 µg/L was reported to have an HQ_ingestion_ and HQ_dermal_ of 0.24 and 0.04 as well as an HQ of 0.29^45^. This was found to be in good agreement with the estimated HQ due to vanadium in the present work which had a maximum HQ value of 0.45.

**Figure 8:**
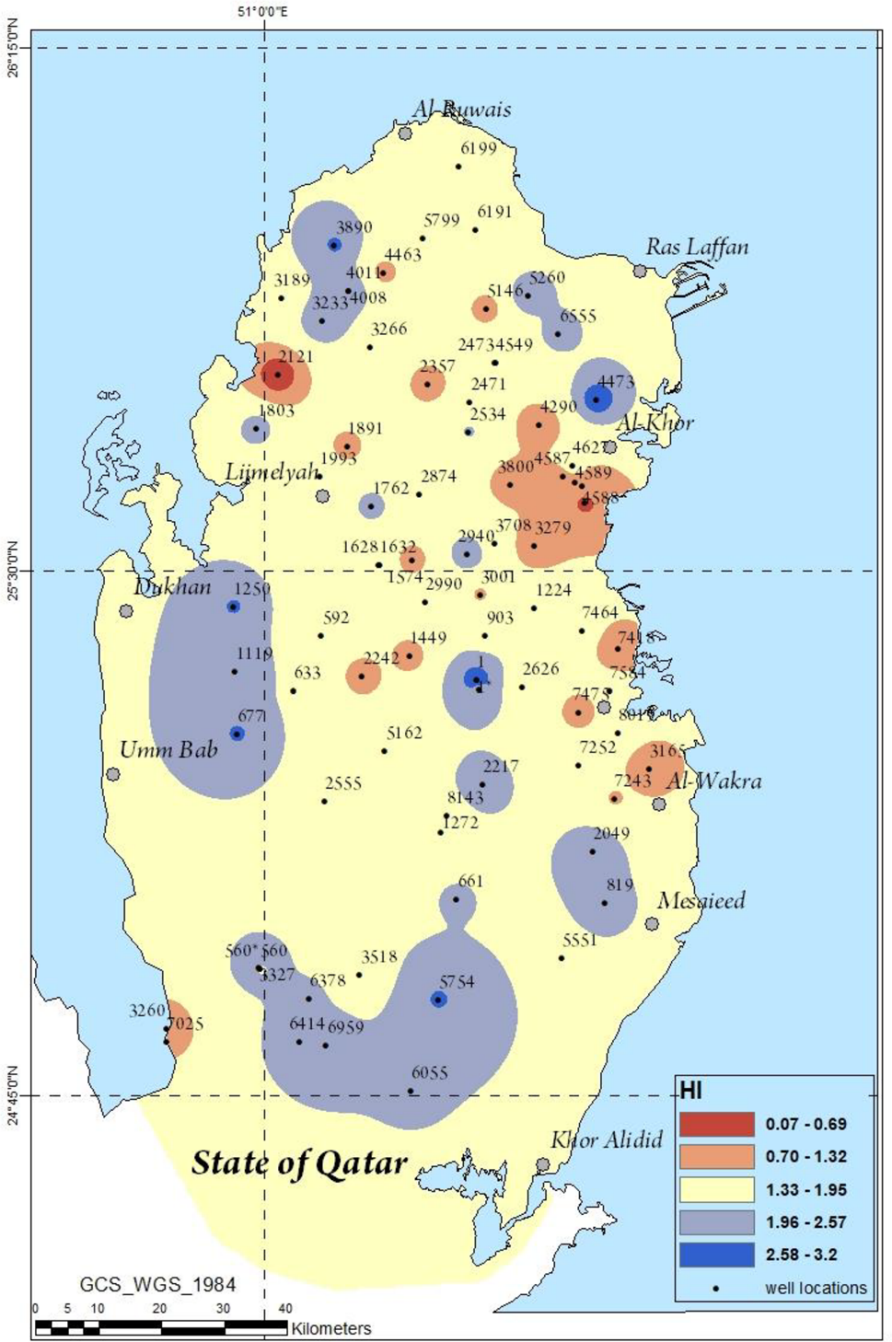
ArcGIS maps depicting the spatial variations in the HI due to the heavy metals in groundwater.

The estimation of the hazard index (HI) for all the studied heavy metals was found to have a range of 0–3.2 whereas the average was 1.6 which is greater than 1. According to US EPA, HI values greater than 1 was reported to pose a potential non-carcinogenic risk to humans. Figure 8 shows a spatial distribution of the HI values calculated for each groundwater sample in the present work. The average contribution of each of the metals in the investigated groundwater samples was observed to account for 0.2, 1.4, 11.3, 32.9 and 42.4% which corresponded to Mn, Mo, Cr, Ag, Sr and V, respectively.

For instance, the investigation of the effect of heavy metals (Mn, Cu, Cd Pb, Cr and Ni) in drinking water on the human health in Nigeria was carried out by Samaila^32^ and found out that the maximum CDI was attributed to Pb with a value of 0.41. Furthermore, the maximum hazard quotient for Pb was reported to be 1030. Moreover, the investigation of the heavy metals (Ni, Se, B, Mn, Al and Zn) in the groundwater water in in China^45^ reported Zn to generate the highest non-carcinogenic human hazard while the reported HQ_Total_ had a range of: 5.9 1 × 10^–3^–7.8 1 × 10^–1^. Moreover, the assessment of the health risks on public due to the contamination of drinking groundwater with heavy metals such as Zn, Ni, Cr, Cd, Mn and Cu in Baghrash Lake Basin which is used for the production of pepper in China was performed by Mamattursun^44^. They reported the non-carcinogenic CDI_Total_ to have a range of 1.9 × 10^–4^–1.9 × 10^–3^ mg/kg/day for adults and 2.15 × 10^–4^–2.2 × 10^–3^ mg/kg/day for children. Moreover, the HQ_Total_ for adults ranged between 6.1 × 10^–3^ and 4.9 × 10^–1^ while that of the children had a range of 7 × 10^–3^–6.7 × 10^–1^. The HI of the investigated groundwater samples was 0.78 for adults and 1.05 for children which was reported to pose risks on children as the HI was greater than 1. The CDI_Total_ and HI at the minimum and average levels in this work was observed to agree with Mamattursun’s work^44^.

### Evaluation of the carcinogenic human health risks

The ILCR value generated from the consumption of groundwater at the existing Cr level reported in the present study can be obtained by first estimating the chronic daily intake of Cr which ranged between 1.4 × 10^–5^ and 1.4 × 10^–3^ mg/kg/day. The ILCR corresponding Cr in the present work was calculated using Eq. (12) keeping in mind the cancer slope factor of Cr of 41 kg/day/mg^32,42^. The ILCR was found to have a range of 5.6 × 10^–4^–5.5 × 10^–2^ with an average of 2.8 × 10^–2^. According to the US EPA, ILCR values between 1 × 10^–3^ and 1 × 10^–1^ is considered at moderate risk level as it will create a cancer risk of 1 in 1000 and 1 in 10. The ILCR in this work was observed to agree with the work cited in literature. For instance, the carcinogenic risks linked with elevated heavy metals levels in drinking water in Iran had a ILCR between 5 × 10^–4^ and 7.6 × 10^–2^ with Cr being the highest contributor with a mean ILCR of 6.5 × 10^–3^^42^. Moreover, the assessment of the heavy metal pollution in 69 groundwater samples in Huaibei Plain farmlands in China showed that 19 samples had ILCR of Cr that was greater than 10^–4^ and that 27.5% of samples had reported health risks associated with them^28^. It was also observed that Cr is the main contributor to human health risks. Likewise, the investigation of the carcinogenic health effects due to heavy metals in drinking water in Nigeria^83^ showed an ILCR of one groundwater source to be 0.14 for adults and 0.16 for children which require urgent attention. The investigation of the human health effect due to the trace metals in the north plain area in China^45^ reported the carcinogenic CDI to have a range of 5.1 × 10^–5^–6.9 × 10^–4^.

## Conclusions

In the present work, a comprehensive overview of the quality of groundwater in Qatar in terms of heavy metals content as well as investigating the cause of effect of the elevation in their levels above the WHO/US-EPA standards was presented. The assessment of the human health risks associated with the existing levels using three of the most used models showed some levels which could be of concern to public health. The chronic daily intake (CDI) of the investigated heavy metals (Ag, Mn, Cr, V, Mo and Sr) through ingestion and dermal pathways had a range of 1.4 × 10^–5^–6.7 × 10^–1^ mg/kg/day while the NCPI’s range was reported at 0–4.39. Moreover, the HI and ILCR were found to have a range of 0–3.2 and 5.6 × 10^–4^–5.5 × 10^–2^, respectively. The assessment of human health risks of groundwater in Qatar in the present work indicated that further investigation must be conducted in order to fully understand the level of contamination and prepare for remediation measures in order to protect public health. This work could be useful for establishing the baseline of heavy metals levels in groundwater of Qatar. This will also help in the determination of any future contamination of groundwater.

### Supplementary Information

Supplementary Figure S1.

## Data Availability

All data relevant to the study are included in the article or uploaded as supplementary information. In addition, the datasets used and/or analyzed during the current study are available from the corresponding author on reasonable request.
