# Supplementary figures and images for: Spatial variations and health risk assessment of heavy metal levels in groundwater of Qatar
Source: Sci Rep. 2024 Jul 10;14:15904. doi: 10.1038/s41598-024-64201-6 (PMC11237053; doi:10.1038/s41598-024-64201-6)

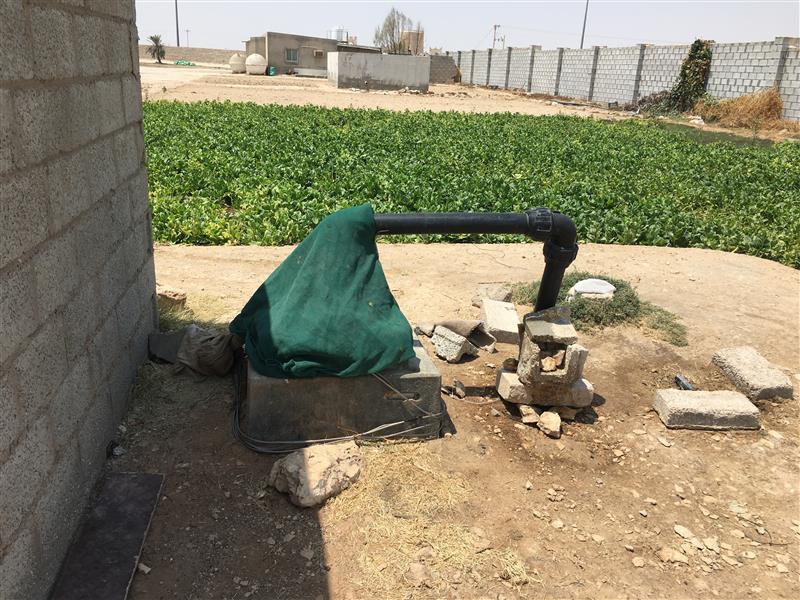

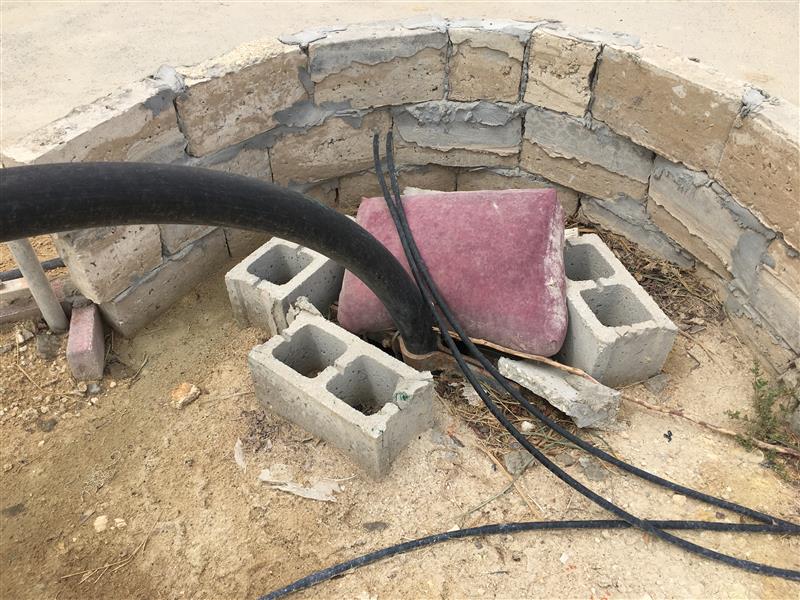


**b**

**a**


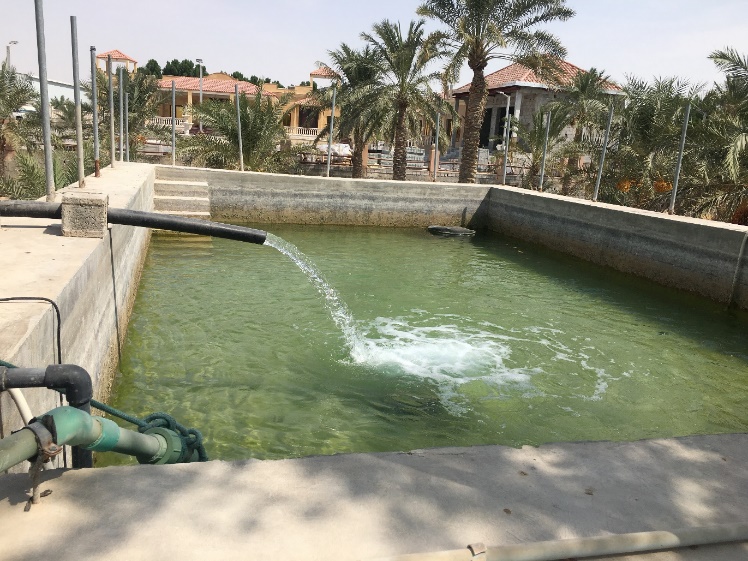


**c**

Figure S1: Groundwater wells (a & b) and water pumped out of the well (c)

Supplement: Supplementary file 1 — Supplementary Figure S1. [file 41598_2024_64201_MOESM1_ESM.docx]
